# Abnormal expression and role of MicroRNA‐214‐3p/SLC8A1 in neonatal Hypoxic‐Ischaemic encephalopathy

**DOI:** 10.1111/iep.12475

**Published:** 2023-04-09

**Authors:** Liu Yang, Li Zhang, Jing Zhu, Yuqian Wang, Ning Zou, Zhengjuan Liu, Yingjie Wang

**Affiliations:** ^1^ Department of Pediatrics The Second Hospital of Dalian Medical University Dalian China

**Keywords:** apoptosis, MiR‐214‐3p, neonatal hypoxic‐Ischaemic encephalopathy, neuronal

## Abstract

Neonatal hypoxic‐ischaemic encephalopathy (HIE) refers to brain damage caused by intra‐uterine distress and asphyxia/hypoxia during the perinatal and neonatal periods. MicroRNA (MiR)‐214‐3p plays a critical role in cell growth and apoptosis. The aim of this study was to investigate the expression and role of miR‐214‐3p in neonatal HIE development, and to explore the underlying mechanisms. The expression of miR‐214‐3p was significantly down‐regulated, while that of *Slc8a1*, a direct target of miR‐214‐3p, was significantly up‐regulated, in the brain tissue of neonatal HIE rats. The over‐expression of miR‐214‐3p promoted the proliferation and inhibited the apoptosis of neurones, while its down‐regulation had the opposite effect. Our results indicate that miR‐214‐3p expression was down‐regulated in neonatal HIE rats, and the up‐regulation of miR‐214‐3p expression protected against HIE development by inhibiting neuronal apoptosis.

## 1 INTRODUCTION

Neonatal hypoxic‐ischaemic encephalopathy (HIE) refers to brain damage caused by intra‐uterine distress and asphyxia/hypoxia during the perinatal and neonatal periods.^1^ HIE can cause nervous system dysfunction, seizures, and motor impairment (cerebral palsy) in infants and young children, seriously threatening children's health and safety, and life, and is the main cause of neonatal death.^2^, ^3^, ^4^ The molecular mechanism of brain injury in infants with HIE is not fully understood. Furthermore, there are no effective satisfactory treatments for HIE.^5^ A better understanding of the pathogenesis of neonatal HIE is crucial for effective clinical management, which is urgently needed.

Recently, increasing evidence has indicated that numerous microRNAs (miRNAs) are involved in the pathogenesis of neurological diseases, including epilepsy, Alzheimer's disease, and ischaemic stroke.^6^, ^7^, ^8^ The relationship between miRNAs and HIE has attracted attention from researchers, and numerous studies have demonstrated the key role of miRNAs in the occurrence and development of HIE.^9^, ^10^, ^11^ MiR‐214‐3p plays a critical role in cell growth and apoptosis.^12^ The expression and role of miR‐214‐3p have been reported in previous studies of cancer.^13^, ^14^, ^15^ MiR‐214‐3p has been shown to be involved in neurological diseases,^16^ and neuronal apoptosis is one of the pathological mechanisms of HIE.^17^, ^18^ The results of these studies indicate that miR‐214‐3p participates in HIE by regulating neuronal apoptosis. However, the expression and role of miRNA‐214‐3p in HIE remain unclear.

Therefore, the aim of this study was to investigate the expression and role of miR‐214‐3p in HIE development, and to explore the underlying molecular mechanisms.

## 2 MATERIALS AND METHODS

### 2.1 Establishment of a neonatal rat HIE model

Perinatal specific pathogen‐free Sprague–Dawley rats were purchased from Liaoning Changsheng Biotechnology Co., Ltd., Liaoning, China (Certificate No. SCXK [Liao] 2020–0001). All experimental procedures were performed in accordance with the Recommended Guidelines for the Care and Use of Laboratory Animals issued by the Chinese Council on Animal Research. The study was approved by the Animal Ethics Committee of The Second Hospital of Dalian Medical University (The Second Hospital of Dalian Medical University College 2017 No. 165). Rat pups were kept in polypropylene cages with nursing mothers and housed at 25 ± 5°C and 50% humidity under 12 h dark/light cycle conditions.

A neonatal rat HIE model was established as previously described.^19^, ^20^ Post‐natal 7‐day‐old (P7) new‐born rats (male and female) were divided into two groups (*n* = 6 rats/group): the control group and the hypoxic‐ischaemic (HI) model group. Briefly, all rats were anaesthetized by isoflurane inhalation and then fixed on the operating table in the supine position. The right common carotid artery was exposed under a dissecting microscope, and the HIE group was permanently ligated with 2.0 sterile‐needle sutures. At both ends of the artery, the blood vessel was cut‐off in the middle of the two ligature points and the wound was sutured. The operation time was 8–10 min. After the operation, the rat awakened and was returned to the mother to recover for 1 h; the rat was then placed in a hypoxic box in a constant temperature water bath set at 37°C. Mixed gas (6% oxygen, 94% nitrogen) was continuously supplied to the box for 2.5 h at a flow rate of 2 L/minute, and the oxygen concentration was maintained at 6%. For rats in the sham operation group, the right common carotid artery was separated without ligation and hypoxia treatment. At 48 h after establishing the HIE model, the rats were anaesthetized by isoflurane inhalation. Then, the chest of the rats was cut open, and the heart was exposed and perfused with normal saline; the brain tissue of the lateral cerebral hemisphere was separated, placed on ice, and stored in an Eppendorf tube at −80°C. The other parts of the rats were perfused with 4% paraformaldehyde after anaesthesia, and the brain tissue was removed and placed in 4% paraformaldehyde for follow‐up experiments. The infarct volume of six rats in each group was measured by 2, 3, 5‐triphenyl‐tetrazolium chloride (TTC) monohydrate (Sigma‐Aldrich) staining, and the cell death rate in the brain tissue was measured by terminal transferase‐mediated DNA end labelling (TUNEL) assay.

### 2.2 Brain infarct size measurement

TTC staining was performed to determine the brain infarct size at 48 h after HIE induction, using a previously described method.^21^ Briefly, serial coronal slices of the rat brain (2‐mm‐thick) were cut. Slices were stained with 2% TTC solution for 5 min at 37°C, and then fixed with 10% formaldehyde overnight. The percentage of the infarct volume in the ipsilateral hemisphere for each slice was scanned and analysed by ImageJ software (National Institutes of Health).

### 2.3 Haematoxylin and eosin staining

Sections were dewaxed with xylene, rehydrated in ethanol, stained with haematoxylin (Solarbio) for 10 min, rinsed with tap water for 30 min, and stained with eosin (Solarbio) for 3 min. After routine dehydration, transparentization, and sealing, the morphology of the infarct zone was observed at 40× magnification using an optical microscope (Olympus).

### 2.4 TUNEL assay

TUNEL staining was performed using an In Situ Cell Death Detection Kit (CAT #11684817910; Roche Applied Bioscience) following the manufacturer's protocol. Briefly, serial coronal slices of the rat brain (5‐μm‐thick) were fixed with 4% paraformaldehyde for 10 min at 20°C, permeabilized with 0.2% Triton X‐100 for 5 min, and then treated with TUNEL reagent for 1 h at 37°C in the dark, according to the manufacturer's instructions. All slides were counter‐stained with Hoechst 33342 (Sigma‐Aldrich). Images were captured using a Zeiss LSM 710 confocal microscope (Wetzlar, Germany), and TUNEL‐positive cells among the total cells were counted in at least 10 randomly selected high‐power fields.

### 2.5 Cell culture and treatment

Primary neurones were isolated and purified as previously described.^22^ Neurones were grown in neurobasal medium supplemented with 2% B27 and 0.5 mM L‐GlutaMax™‐I (Gibco, Grand Island), and incubated in a humidified atmosphere with 5% carbon dioxide at 37°C.

An oxygen and glucose deprivation (OGD) model of primary neurones was established as described previously.^23^ For OGD induction, the cell culture medium was replaced with glucose‐free Dulbecco's Modified Eagle Medium (Gibco). The primary neurones were divided into six groups: control group (untreated primary neurones), OGD group (untreated primary neurones subjected to OGD induction), mimic control group (negative control with miR‐214‐3p mimic, NCM [primary neurones transfected with mimic control for 48 h and then subjected to OGD induction]), inhibitor control group (negative control with miR‐214‐3p inhibitor, NCI [primary neurones transfected with inhibitor control for 48 h and then subjected to OGD induction]), mimic group (primary neurones transfected with miR‐214‐3p mimic control for 48 h and then subjected to OGD induction), and inhibitor group (primary neurones transfected with miR‐214‐3p inhibitor control for 48 h and then subjected to OGD induction).

### 2.6 Cell transfection

The miR‐214‐3p mimic/inhibitor and negative control were obtained from Shanghai GenePharma Co., Ltd., Shanghai, China. For cell transfection, primary neurones were plated into a 6‐well plate (approximately 1 × 10^6^ cells/well) and cultured at 37°C for 24 h. Cells were transfected with the miR‐214‐3p inhibitor (5′‐ACUGCCUGUCUGUGCCUGCUGU‐3′), negative control of the miR‐214‐3p inhibitor (5′‐CAGUACUUUUGUGUAGUACAA‐3′), miR‐214‐3p mimic (sense: 5′‐ACAGCAGGCACAGACAGGCAGU‐3′; anti‐sense: 5′‐UGCCUGUCUGUGCCUGCUGUUU‐3′), and negative control of the miR‐214‐3p mimic (sense: 5′‐UUCUCCGAACGUGUCACGUTT‐3′; anti‐sense: 5′‐ACGUGACACGUUCGGAGAATT‐3′) at a final concentration of 50 nM using Lipofectamine 2000 reagent (Invitrogen) following the manufacturer's instructions. The transfection efficiency was measured after 48 h.

### 2.7 Dual luciferase reporter assay

TargetScan (http://www.targetscan.org/vert_71) was used to predict the targets of miR‐214‐3p, and *Slc8a1* was identified as a potential target of miR‐214‐3p. To confirm the direct binding sites between miR‐214‐3p and *Slc8a1*, a dual luciferase reporter assay was performed. The 3′‐untranslated region (UTR) of wild‐type and mutant *Slc8a1* was cloned into a pmiR‐RB‐REPORT dual‐luciferase reporter vector (Guangzhou RiboBio Co., Ltd.) according to the manufacturer's instructions. Cells were co‐transfected with wild‐type or mutant *Slc8a1* and the miR‐214‐3p mimic or 50 nM mimic control following the manufacturer's protocol. After 48 h, luciferase activity was assessed using the Dual‐Luciferase Assay System (Promega Corporation) following the manufacturer's protocol. Luciferase activity detected in the cells was normalized to *Renilla* luciferase activity.

### 2.8 3‐[4, 5‐Dimethylthiazole‐2‐yl]‐2, 5‐Diphenyltetrazolium bromide (MTT) assay

Cell viability was measured by performing an MTT assay. After treating the cells as described above, neurones (approximately 1 × 10^4^ cells/well) were seeded into 96‐well plates and cultured for 24, 48, or 72 h. Subsequently, 20 μL MTT solution (0.5 mg/mL; Sigma‐Aldrich) was added to each well, and the cells were incubated at 37°C for another 4 h. Cell viability was assessed by measuring the absorbance at 570 nm using a FLUOstar Omega microplate reader (BMG Labtech).

### 2.9 Flow cytometry

To analyse cell apoptosis, we used an Annexin V‐Fluorescein Isothiocyanate Cell Apoptosis Detection Kit (CAT #C1062S; Beyotime Institute of Biotechnology). After treatment, the cells were harvested using 0.25% Trypsin, washed with phosphate‐buffered saline, and exposed to 5 μL Annexin V‐fluorescein isothiocyanate and 5 μL propidium iodide for 30 min in the dark at room temperature. A flow cytometer (BD Biosciences) was used to analyse cell apoptosis. Each test was performed in triplicate.

### 2.10 Western blotting

Western blotting was conducted to measure the protein levels. Radioimmunoprecipitation assay buffer (Beyotime Institute of Biotechnology) was used to extract proteins from the primary neurones and brain tissues of six rats in each group according to the manufacturer's instructions. Protein concentrations were assessed using a Bicinchoninic Acid Assay Kit (Pierce Biotechnology). Equal amounts of protein samples (30 μg per lane) were separated on 12% sodium dodecyl sulphate‐polyacrylamide gels and transferred onto polyvinylidene fluoride membranes (Merck Millipore). After blocking with 5% skim milk at room temperature for 1 h, the membranes were incubated overnight at 4°C with primary antibodies (rabbit anti‐Bcl‐2: CAT #3498, 1:1000, Cell Signalling Technology, Danvers, MA, USA; rabbit anti‐caspase‐3: CAT #9662, 1:1000, Cell Signalling Technology; rabbit anti‐NSE: CAT #AF5473, 1:1000, Affinity Biosciences, Jiangsu, China; rabbit anti‐S100B: CAT #9550, 1:1000, Cell Signalling Technology; and rabbit anti‐SLC8A1 (NCX): CAT #79350, 1:1000, Cell Signalling Technology). Membranes were then incubated with the secondary antibody (anti‐rabbit IgG horseradish peroxidase‐linked antibody: CAT #7074, 1:2000, Cell Signalling Technology) at room temperature for 2 h. Protein bands were visualized using an electrochemiluminescence Western Blotting Detection Kit (Merck Millipore).

### 2.11 Quantitative reverse‐transcription polymerase chain reaction (qRT‐PCR)

Total RNA was extracted from brain tissues and cells using TRIzol reagent (Invitrogen) according to the manufacturer's instructions. We reverse‐transcribed RNA into complementary DNA using a TaqMan MicroRNA Reverse Transcription Kit (Applied Biosystems) following the manufacturer's protocol. To analyse the synthesized complementary DNA, qRT‐PCR was performed using a SYBR Premix Ex TaqTM II (TliRNaseH Plus) Kit (Takara Bio) according to the manufacturer's instructions. *U6* or *Gapdh* was used as an internal control. The primer sequences used for qRT‐PCR were as follows:

miR‐214‐3p forward: 5′‐GACAGCAGGCACAGACA‐3′.

miR‐214‐3p reverse: 5′‐GTGCAGGGTCCGAGG‐3′.

*Slc8a1* forward: 5′‐ACAACATGCGGCGATTAAGTC‐3′.

*Slc8a1* reverse: 5′‐GCTCTAGCAATTTTGTCCCCA‐3′.

*Bcl‐2* forward: 5′‐TTGGATCAGGG AGTTGGAAG‐3′.

*Bcl‐2* reverse: 5′‐TGTCCCTACCAACCAGAAGG‐3′.

*Caspase‐3* forward: 5′‐AGAACTGGACTGTGGCATTG‐3′.

*Caspase‐3* reverse: 5′‐CACAAAGCGACTGGATGAAC‐3′.

*Gapdh* forward: 5′‐GGAGCGAGATCCCTCCAAAAT‐3′.

*Gapdh* reverse: 5′‐GGCTGTTGTCATACTTCTCATGG‐3′.

*U6* forward: 5′‐GGCAAGGATGTAGTCGTGGAACTC‐3′.

*U6* reverse: 5′‐GGTCTGTGACACTGATGTCGGTTAG‐3′.

The relative messenger RNA (mRNA) expression levels were calculated using the 2^−ΔΔCT^ method.^24^

### 2.12 Statistical analyses

All experiments were performed at least three times. Statistical analysis was performed using spss 17.0 (spss Inc). Data were expressed as mean ± standard deviation (SD). Differences between groups were analysed by one‐way analysis of variance with Tukey's post‐hoc test or Student's *t*‐test. A *p*‐value < .05 was considered statistically significant.

## 3 RESULTS

### 3.1 HIE‐exacerbated brain injury and neuronal apoptosis

**FIGURE 1:**
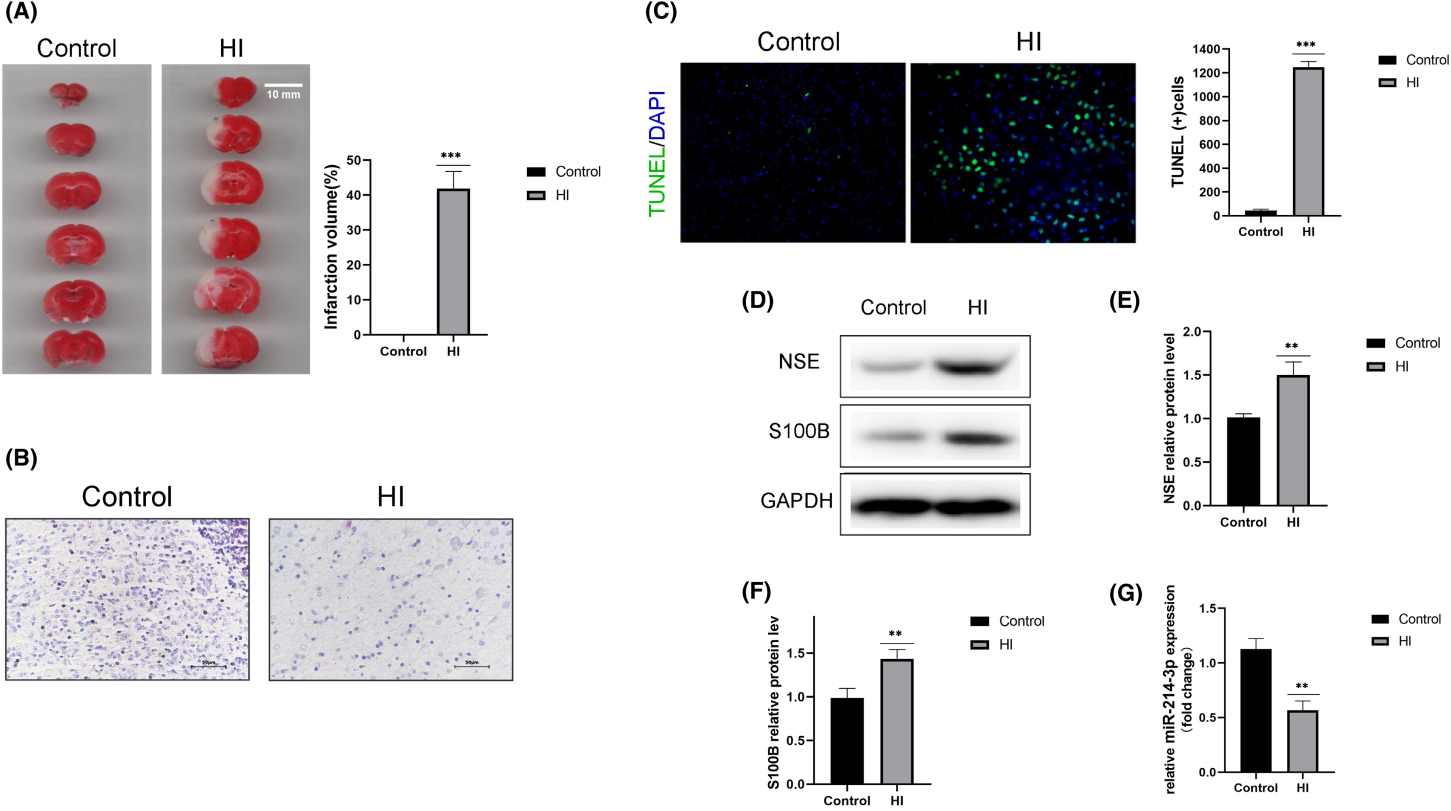
Hypoxic‐Ischaemic Encephalopathy (HIE) Exacerbated Brain Injury and Neuronal Apoptosis. (A) HIE caused severe infarcts in rat brains, as shown by 2, 3, 5‐triphenyl‐tetrazolium chloride (TTC) staining. (B) Haematoxylin and eosin staining in the infarct zone after hypoxic‐ischaemic (HI) injury (original magnification, ×400). (C) Cell apoptosis in brain tissue analysed using the terminal transferase‐mediated DNA end labelling (TUNEL) assay (original magnification, ×200) (TUNEL [green]/4′, 6‐diamidino‐2‐phenylindole [DAPI] [blue]). (D–F) Western blotting detected NSE and S100B protein levels in the brain tissue of HIE rats. (G) MiR‐214‐3p levels in the brain tissue of HIE rats measured by quantitative reverse‐transcription polymerase chain reaction. Data are expressed as mean ± standard deviation. **p < .01, ***p < .001 vs. control (*n* = 6 rats/group).

The extent of brain injury was evaluated by calculating the infarct volume and detecting cell apoptosis, as well as brain‐related biomarkers, including NSE and S100B. TTC staining showed that HIE caused severe infarcts in rat brains (Figure 1A). Compared with the control group, the white matter in the HIE group was lightly stained, the number of cells was reduced, and the structure was sparse, presenting a mesh‐like change (Figure 1B). Cell apoptosis in brain tissue was investigated using TUNEL assay. The percentage of TUNEL‐positive cells increased considerably in the HIE group compared to the control group (Figure 1C). Western blotting revealed that NSE and S100B protein levels were significantly higher in the brain tissue of HIE rats than in control rats (Figure 1D–F). However, miR‐214‐3p levels significantly decreased in the brain tissue of HIE rats compared with controls (Figure 1G).

### 3.2 OGD‐induced neuronal injury

**FIGURE 2:**
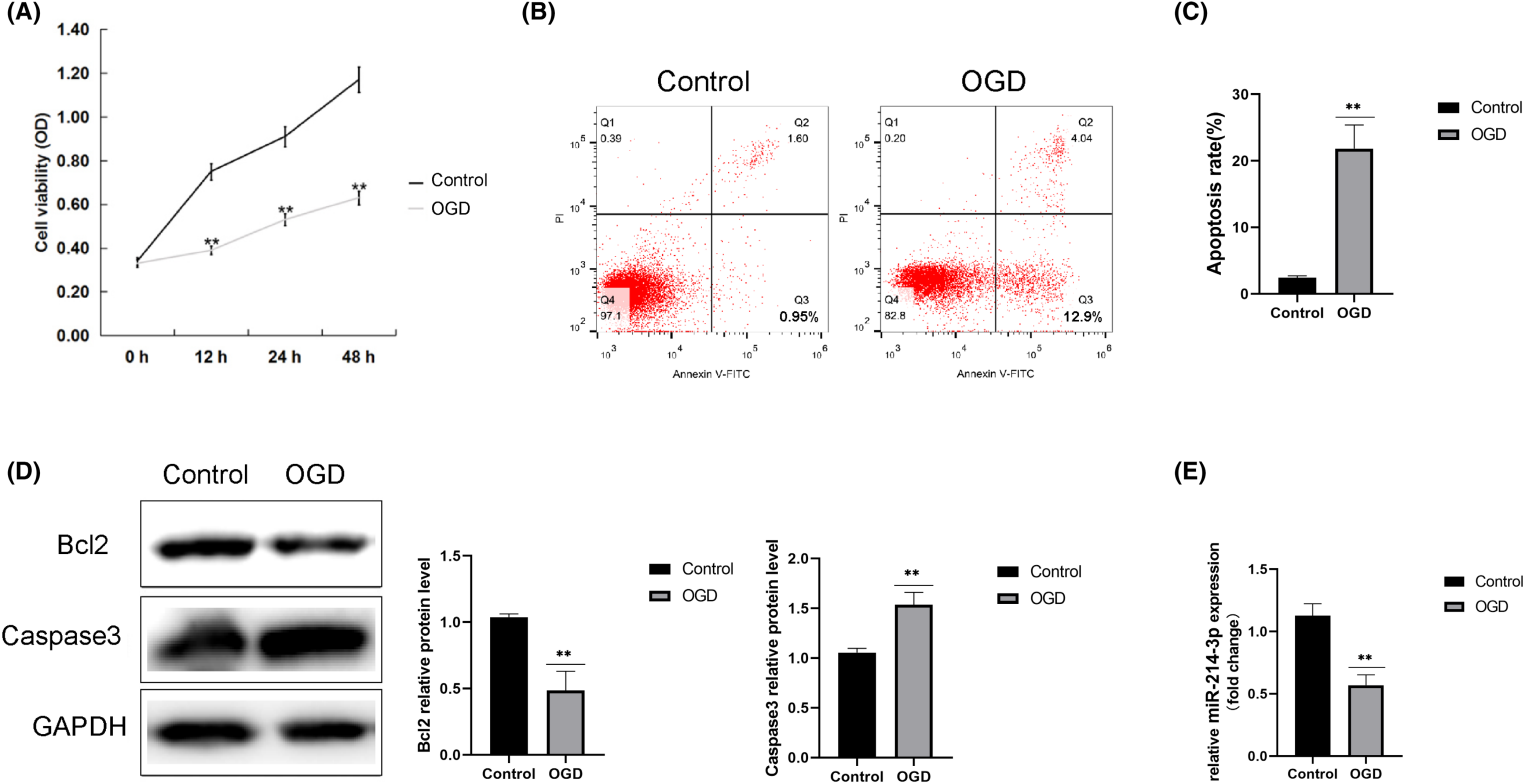
Oxygen–Glucose Deprivation (OGD)‐Induced Neuronal Injury. An OGD model of primary neurones was developed. (A) Cell viability was determined by 3‐[4, 5‐dimethylthiazole‐2‐yl]‐2, 5‐diphenyltetrazolium bromide assay (MTT). (B,C) Cell apoptosis was analysed by flow cytometry, and the apoptosis rate was calculated. (D) Protein levels of Bcl‐2 and Caspase‐3 were detected using western blotting. (E) MiR‐214‐3p levels in the neurones were measured using quantitative reverse‐transcription polymerase chain reaction. Data are expressed as mean ± standard deviation. **p < .01, ***p < .001 vs. control (*n* = 6 rats/group).

An OGD model of primary neurones was developed according to a previous study.^23^ Results indicated that OGD treatment significantly inhibited neuronal viability (Figure 2A), induced neuronal apoptosis (Figure 2B,C), enhanced Caspase‐3 protein levels, and reduced Bcl‐2 protein levels (Figure 2D). As expected, OGD significantly decreased miR‐214‐3p levels in neurones (Figure 2E).

### 3.3 MiR‐214‐3p promoted OGD‐induced neuronal viability

**FIGURE 3:**
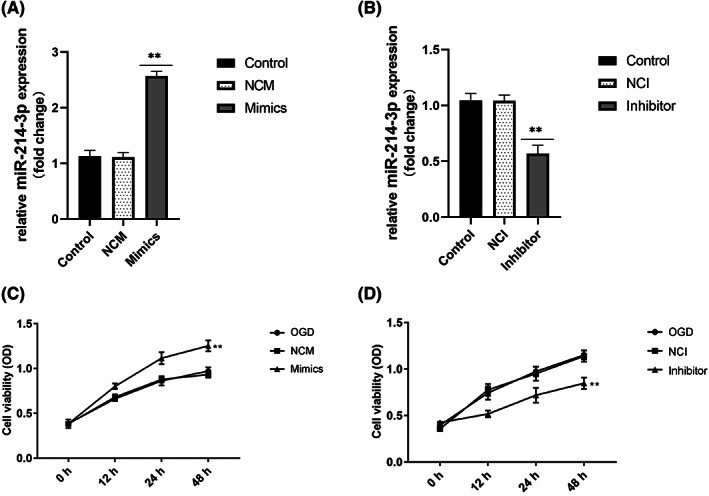
Effect of MiR‐214‐3p on Oxygen–Glucose Deprivation (OGD)‐Induced Neuronal Viability. (A,B) Levels of miR‐214‐3p in the neurones of different groups were measured by quantitative reverse‐transcription polymerase chain reaction. (C,D) Cell viability was determined by 3‐[4, 5‐dimethyl‐thiazole‐2‐yl]‐2, 5‐diphenyltetrazolium bromide (MTT) assay. Data are expressed as mean ± standard deviation. **p < .01 vs. control or OGD (*n* = 6 rats/group). NCI, inhibitor control group; NCM, mimic control group.

To determine the effect of miR‐214‐3p on HIE brain injury, the miR‐214‐3p mimic or miR‐214‐3p inhibitor was transfected into primary neurones for 48 h; thereafter, cells were subjected to OGD. As shown in Figure 3A and Figure 3B, compared with the untreated cells, miR‐214‐3p expression was significantly up‐regulated in cells transfected with the miR‐214‐3p mimic and down‐regulated in cells transfected with the miR‐214‐3p inhibitor, confirming successful preparation. In addition, compared with the OGD group, transfection of the miR‐214‐3p mimic for 48 h significantly promoted OGD‐induced neuronal viability (Figure 3C), while transfection with the miR‐214‐3p inhibitor for 48 h significantly inhibited OGD‐induced neuronal viability (Figure 3D). These results demonstrated that miR‐214‐3p could promote OGD‐induced neuronal viability.

### 3.4 MiR‐214‐3p inhibited OGD‐induced neuronal apoptosis

**FIGURE 4:**
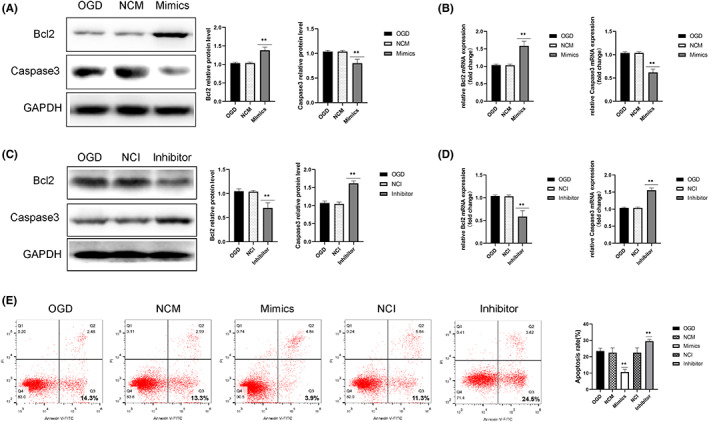
Effect of MiR‐214‐3p on Oxygen–Glucose Deprivation (OGD)‐Induced Neuronal Apoptosis. (A, C) Protein levels of Bcl‐2 and Caspase‐3 in the neurones of different groups. (B, D) Messenger RNA levels of *Bcl‐2* and *Caspase‐3* in the neurones of different groups. (E) Cell apoptosis was analysed by flow cytometry, and the apoptosis rate was calculated. Data are expressed as mean ± standard deviation. **p < .01 vs. OGD (*n* = 6 rats/group). NCI, inhibitor control group; NCM, mimic control group.

To further explore the role of miR‐214‐3p in the regulation of neuronal apoptosis after OGD exposure, apoptosis‐related protein levels were determined. As shown in Figure 4A–D, treatment with the miR‐214‐3p mimic significantly increased the protein and mRNA levels of Bcl‐2/*Bcl‐2*, and decreased the protein and mRNA levels of Caspase‐3/*Caspase‐3*, whereas the miR‐214‐3p inhibitor significantly decreased the protein and mRNA levels of Bcl‐2/*Bcl‐2*, and increased the protein and mRNA levels of Caspase‐3/*Caspase‐3*, compared with those in OGD group. In addition, flow cytometry indicated that the levels of neuronal apoptosis in the miR‐214‐3p mimic‐transfected group were lower than those in the OGD group, whereas the levels of neuronal apoptosis in the miR‐214‐3p inhibitor‐transfected group were higher than those in the OGD group (Figure 4E). These results demonstrated that miR‐214‐3p could inhibit OGD‐induced neuronal apoptosis.

### 3.5 Slc8a1 was a direct target of MiR‐214‐3p

**FIGURE 5:**
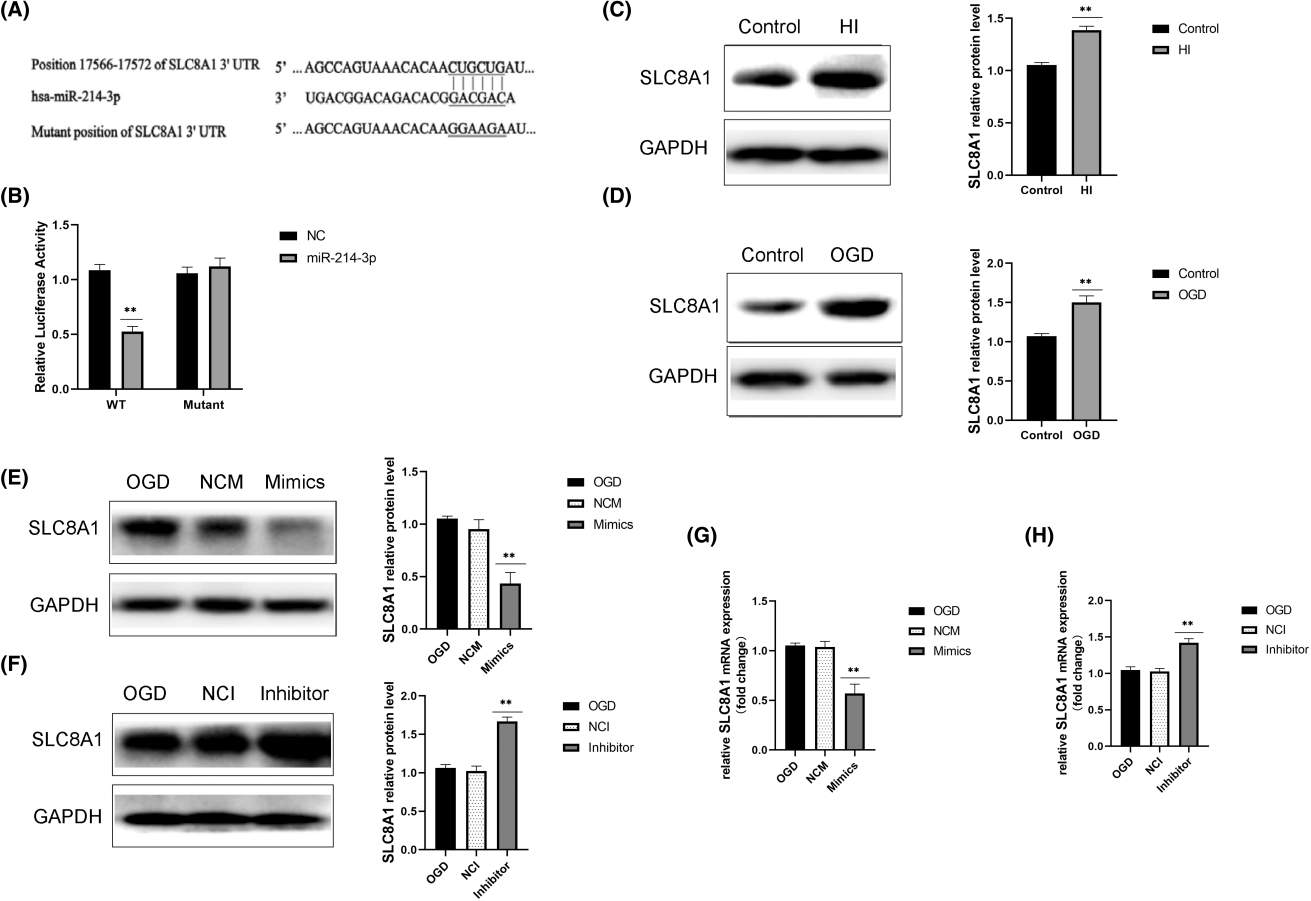
Relationship Between MiR‐214‐3p and SLC8A1. (A) Interaction between miR‐214‐3p and the 3′‐untranslated region (UTR) of *Slc8a1*. (B) Luciferase activity of a reporter containing the wild‐type/mutant *Slc8a1* 3′‐UTR. ‘MUT‐SLC8A1’ indicates the *Slc8a1* 3′‐UTR with a mutation in the miR‐214‐3p binding site. Data are expressed as mean ± standard deviation of three independent experiments. **p < .01 vs. control (NC). (C) SLC8A1 protein levels in the brain tissue of hypoxic‐ischaemic encephalopathy (HIE) rats, as detected by western blotting. (D) SLC8A1 protein levels determined by western blotting in oxygen–glucose deprivation (OGD)‐treated neurones. (E, F) SLC8A1 protein levels determined by western blotting in OGD‐treated neurones in different groups. (G,H) *Slc8a1* messenger RNA levels measured by quantitative reverse‐transcription polymerase chain reaction in OGD‐treated neurones in different groups. Data are expressed as mean ± standard deviation. **p < .01 vs. control or OGD (*n* = 6 rats/group). NCI, inhibitor control group; NCM, mimic control group.

The binding sites between miR‐214‐3p and the 3′‐UTR of *Slc8a1* were predicted by TargetScan (Figure 5A), and the prediction was confirmed by a dual luciferase reporter assay. As shown in Figure 5B, the miR‐214‐3p mimic reduced luciferase activity in neurones co‐transfected with wild‐type *Slc8a1*. Conversely, the miR‐214‐3p mimic had no significant effect on the luciferase activity in neurones co‐transfected with mutant *Slc8a1*. In addition, the protein levels of SLC8A1 were down‐regulated in the brain tissue of neonatal HIE rats compared with controls (Figure 5C). OGD significantly increased the protein levels of SLC8A1 in neurones (Figure 5D). Furthermore, to confirm the role of miR‐214‐3p in the regulation of *Slc8a1* expression, both gain and loss of miR‐214‐3p function were tested by transfecting neurones with a miR‐214‐3p mimic/inhibitor. As shown in Figure 5E and Figure 5G, the miR‐214‐3p mimic effectively decreased the protein and mRNA expression of SLC8A1/*Slc8a1*, whereas the miR‐214‐3p inhibitor increased (Figure 5F,H) the protein and mRNA expression of SLC8A1/*Slc8a1* in rat neurones after OGD.

## 4 DISCUSSION

Although HIE involves neonatal brain injury caused by perinatal asphyxia,^25^ the mechanism underlying neonatal HIE‐mediated brain injury is not fully understood. Accurate detection of body fluid biomarkers in neonatal HIE is important for implementing early interventions to reduce neonatal mortality and morbidity, and the degree of disability. In this study, we demonstrated that HIE exacerbated brain injury and neuronal apoptosis. In addition, miR‐214‐3p expression was significantly decreased in the brain tissue of neonatal HIE rats, while *Slc8a1* expression was significantly increased. Our bioinformatics analysis revealed that *Slc8a1* is a direct target of miR‐214‐3p. In addition, miR‐214‐3p promoted proliferation and inhibited the apoptosis of OGD‐treated neurones. Taken together, our findings suggest that miR‐214‐3p is a potential therapeutic target for neonatal HIE.

NSE, a glycolytic enolase enzyme, is present in the cytoplasm of cells with neuroendocrine differentiation and neurones in the brain. NSE levels can be indirectly estimated to determine the degree of neuronal damage and prognosis of neonatal HIE.^26^, ^27^, ^28^ S100B—an acidic calcium‐binding protein in nervous tissues—is a potential biomarker to determine and evaluate minor brain damage of neonatal HIE.^28^, ^29^ Our results showed that the levels of brain injury‐related biomarkers, namely NSE and S100B, were increased in the brain tissue of neonatal HIE rats, indicating that HIE induced brain injury.

MiRNAs in the brain are closely related to the occurrence and development of HIE.^30^, ^31^, ^32^ MiR‐214‐3p, which has been studied extensively as a pathway in the development of cancer,^14^, ^15^, ^33^, ^34^, ^35^, ^36^and has been shown to play a key role in regulating cell growth and apoptosis.^13^, ^14^, ^15^ These studies have reported a strong correlation between miR‐214‐3p levels and cell survival. MiR‐214‐3p is also closely related to tissue hypoxic‐ischaemic injury. Studies have shown that miR‐214‐3p levels significantly decreased in hypoxia‐induced cardiomyocyte injury, and PVT1 sponged miR‐214‐3p to regulate hypoxia‐induced cardiomyocyte injury.^37^ In addition, miR‐214‐3p regulates hypoxia‐mediated proliferation and pulmonary artery smooth muscle cell migration by targeting ARHGEF12.^38^ A recent study showed that hypoxia significantly up‐regulated the expression of miR‐214‐3p in HK‐2 cells, in unilateral ureteral obstructive nephropathy, and in patients with chronic kidney disease, and that miR‐214‐3p knock‐down reversed the hypoxia‐induced interstitial transformation of renal tubular epithelial cells and ameliorated fibrosis.^39^ In this study, miR‐214‐3p expression was found to be down‐regulated in the brain tissue of neonatal HIE rats. MiR‐214‐3p also plays a critical role in preventing neuronal apoptosis and inhibiting neuronal pyroptosis and autophagy.^40^, ^41^ However, our target analysis identified specific pathways and biological processes that may be affected by or linked to HIE‐associated neurological injury. HIE induction led to excessive neuronal loss consistent with the results of a previous study.^40^ Furthermore, miR‐214‐3p promoted cell proliferation and inhibited the apoptosis of OGD‐treated neurones,^15^ which is also consistent with the results of a previous study.^14^ Tetra‐methylpyrazine reportedly alleviates neuronal apoptosis in injured spinal cord by down‐regulating miR‐214‐3p expression.^42^ Western blotting showed that caspase‐3 levels decreased, and Bcl‐2 levels increased, in neurones overexpressing miR‐214‐3p. Conversely, caspase‐3 levels decreased, and Bcl‐2 levels increased, in neurones with low expression of miR‐214‐3p.

Finally, to determine the molecular mechanism underlying the role of miR‐214‐3p in neuronal apoptosis, we used TargetScan to predict potential targets of miR‐214‐3p. We identified hundreds of potential targets of miR‐214‐3p, including *Slc8a1*. *Slc8a1*, also known as *Ncx1*, plays a critical role in the generation and control of calcium signals, and is involved in the regulation of cell apoptosis.^43^, ^44^ Interestingly, the expression and role of *Slc8a1* in HIE remain largely unclear. Thus, we selected *Slc8a1* for further investigation. Our results confirmed that *Slc8a1* was a direct target of miR‐214‐3p. *Slc8a1* expression was up‐regulated in HIE rats and OGD‐treated neurones, and it was negatively regulated by miR‐214‐3p.

In conclusion, we demonstrated that miR‐214‐3p expression was down‐regulated in HIE using in vitro and in vivo models, and that it could promote neuronal proliferation and inhibit neuronal apoptosis in OGD‐treated neurones. *Slc8a1* is a target of miR‐214‐3p. These results indicate the potential of miR‐214‐3p to serve as a therapeutic target for future treatments of neonatal HIE. However, this is still only a preliminary study highlighting the role of miR‐214‐3p in HIE. To further clarify the role of miR‐214‐3p in HIE, to confirm whether miR‐214‐3p affects neurones and regulates apoptosis‐related protein expression (Caspase‐3 and Bcl‐2) in neurones by directly targeting *Slc8a1*, and to clarify the exact mechanism by which miR‐214‐3p inhibits neuronal apoptosis in HIE in vivo, further research is required. We will investigate these aspects in the future.

## AUTHOR CONTRIBUTIONS

**Liu Yang:** Conceptualization, Methodology, Software, Data curation, Writing – original draft. **Li Zhang:** Data curation, Writing – original draft. **Jing Zhu:** Visualization, Investigation, Writing – review & editing. **Yuqian Wang:** Visualization, Investigation, Writing – review & editing. **Ning Zou:** Supervision. **Zhengjuan Liu:** Supervision. **Yingjie Wang:** Software, Validation, Writing – review & editing. All authors have read and approved the final manuscript.

## FUNDING INFORMATION

This work was supported by grants from the National Natural Science Foundation of China [grant numbers 82201896], the Basic scientific research project of Liaoning Provincial Department of Education [grant number LJKMZ20221294].

## CONFLICT OF INTEREST STATEMENT

The authors declare that there are no conflicts of interest.
